# AlphaMissenseR: an integrated framework for investigating missense mutations in human protein-coding genes

**DOI:** 10.1093/bioadv/vbaf093

**Published:** 2025-04-23

**Authors:** Tram N Nguyen, Tyrone Lee, Nitesh Turaga, Robert Gentleman, Ludwig Geistlinger, Martin Morgan

**Affiliations:** Center for Computational Biomedicine, Harvard Medical School, Boston, MA 02115, United States; Center for Computational Biomedicine, Harvard Medical School, Boston, MA 02115, United States; Center for Computational Biomedicine, Harvard Medical School, Boston, MA 02115, United States; Center for Computational Biomedicine, Harvard Medical School, Boston, MA 02115, United States; Dana Farber Cancer Institute, Harvard School of Public Health, Boston, MA 02115, United States; Center for Computational Biomedicine, Harvard Medical School, Boston, MA 02115, United States; Roswell Park Comprehensive Cancer Center, Buffalo, NY 14221, United States

## Abstract

**Summary:**

AlphaMissense is an AI model from Google DeepMind that predicts the pathogenicity of every possible missense mutation in the human proteome. We present AlphaMissenseR, an R/Bioconductor package that facilitates performant and reproducible access to these predictions and that provides functionality for analysis, visualization, validation, and benchmarking. AlphaMissenseR integrates with Bioconductor facilities for genomic region analysis, and provides multi-level visualization and interactive exploration of variant pathogenicity in a genome browser and on 3D protein structures. In addition, AlphaMissenseR integrates with major clinical and experimental variant databases for contrasting predicted and clinically derived pathogenicity scores, and for systematic benchmarking of existing and new variant effect prediction methods across a large collection of deep mutational scanning assays.

**Availability and implementation:**

AlphaMissense data resources are distributed under the CC-BY 4.0 license and the AlphaMissenseR package is available from Bioconductor (https://bioconductor.org/packages/AlphaMissenseR) under the Artistic 2.0 license.

## 1 Introduction

**Figure 1.**
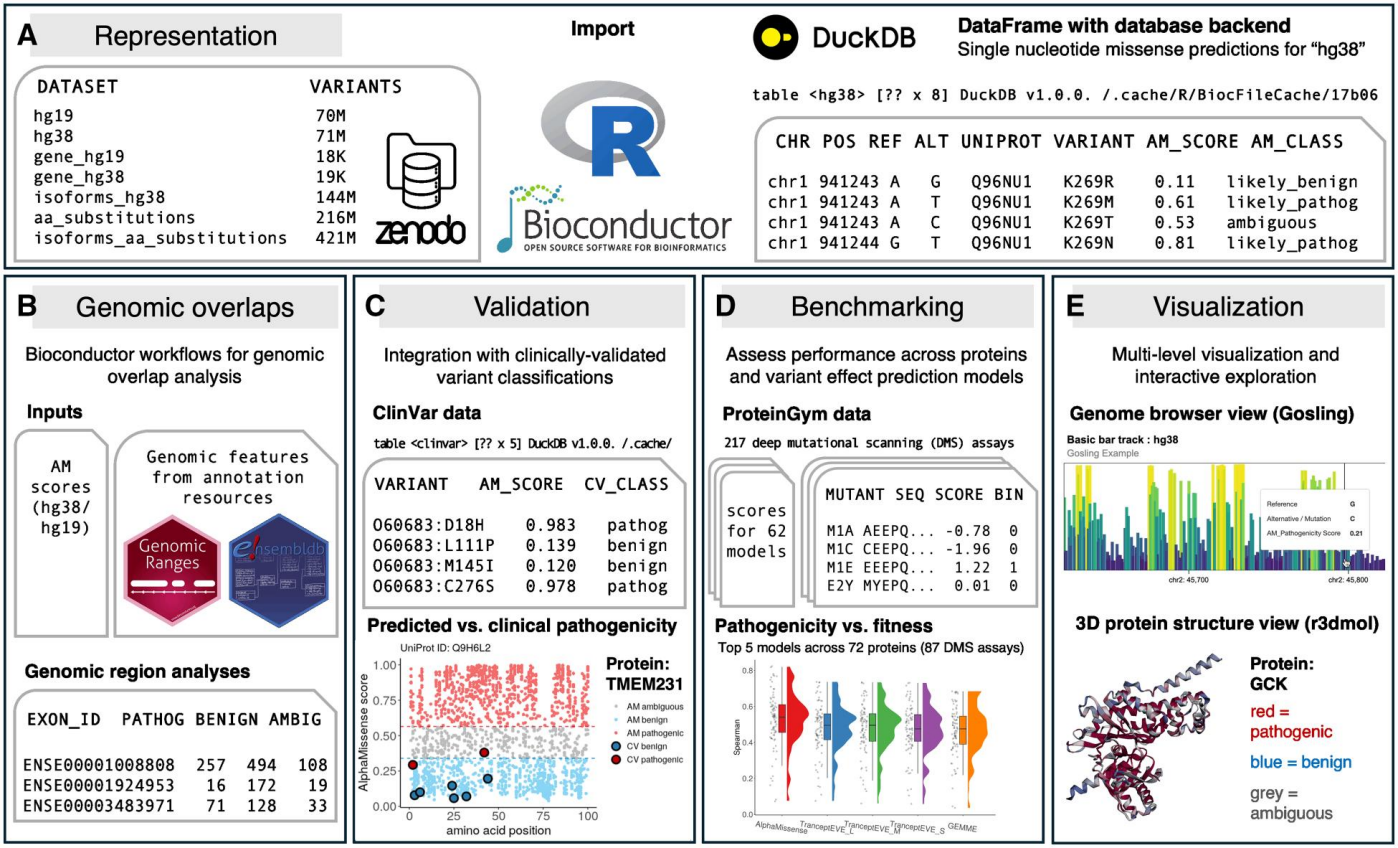
Overview. (A) The AlphaMissenseR package imports AlphaMissense datasets hosted on Zenodo into R using a DuckDB database as backend. The package provides functionality for integration with data resources and analysis capabilities for: (B) genomic region analysis with Bioconductor, (C) contrasting AlphaMissense predictions with clinical evidence from ClinVar (Landrum *et al.* 2018), (D) benchmarking of AlphaMissense against other methods on experimental fitness assays from ProteinGym (Notin *et al.* 2023), and (E) visualization and interactive exploration of variant pathogenicity on gene and protein level.

Missense mutations are genetic variants that alter the amino acid sequence of proteins, potentially disrupting their structure and function (Zhang *et al.* 2012). Benign variants have no or only limited effect on protein fitness and typically do not have physiological consequences, while pathogenic variants cause a strong reduction in fitness and confer a risk of developing a certain genetic disorder or disease (Karczewski *et al.* 2020). Despite the significance of missense variants for human health and disease, comprehensively classifying their effects is an ongoing challenge (Karczewski *et al.* 2020). Databases such as ClinVar (Landrum *et al.* 2018) provide clinically validated pathogenicity classifications for >80k variants, yet the vast majority of possible missense variants in the human genome remain unclassified. To address this challenge, Google DeepMind developed AlphaMissense, an AI model that predicts the pathogenicity of every possible missense mutation in the human proteome (Cheng *et al.* 2023). The model builds on the protein structure prediction tool AlphaFold2 (Jumper *et al.* 2021) and uses predicted structural context and fine-tuning on weak labels from population frequency data to provide variant effect predictions at scale. Cheng *et al.* (2023) applied AlphaMissense to obtain genome-wide, gene-level aggregate, and proteome-wide predictions and made them available to the community under the CC-BY 4.0 license on Zenodo (Fig. 1A). The data release includes: (i) predictions for all possible single nucleotide missense variants in 19k canonical protein-coding transcripts (hg19 and hg38) and 60k noncanonical transcript isoforms (hg38, GENCODE V32), (ii) gene-level summaries providing average pathogenicity scores for canonical protein-coding transcripts (hg19 and hg38), and (iii) predictions for all theoretically possible single amino acid substitutions in canonical human proteins (216M) and noncanonical isoforms (421M).

Here, we describe performant and reproducible access to the AlphaMissense community resources through the AlphaMissenseR R/Bioconductor package, and integration with tools and data resources for analysis, visualization, validation, and benchmarking. Taken together, AlphaMissenseR enables targeted investigations of missense mutations in genomic features of interest, and implements an extensible framework for the systematic and reproducible benchmarking of new and existing variant effect prediction tools.

## 2 Features

### 2.1 Import and representation of AlphaMissense data

AlphaMissenseR reads the AlphaMissense community resources from Zenodo into R using a DuckDB (Raasveldt and Mühleisen 2019) database-backend for efficient querying and analysis with a minimal memory footprint (Fig. 1A). Files are downloaded once and subsequently cached using BiocFileCache (Shepherd and Morgan 2024), and the data can easily be manipulated using tidy data management grammar (Wickham *et al.* 2019).

### 2.2 Overlap analysis with functional genomic regions

Once imported into R, AlphaMissenseR provides seamless interfacing with Bioconductor facilities for genomic region analysis (Fig. 1B). This includes memory-efficient representation and manipulation of single nucleotide variant data in designated data structures from the GenomicRanges package (Lawrence *et al.* 2013), and overlap analysis with functional genomic regions defined in genome annotation packages such as the ensembldb package (Rainer *et al.* 2019) or resources available through AnnotationHub (Morgan and Shepherd 2024). The storage of AlphaMissense data in GenomicRanges data structures provides access to an extensive set of algebraic operations for the analysis of genomic regions and interoperability with a wide range of domain-specific packages from the Bioconductor ecosystem. This also includes integration with genome annotation resources, which facilitates the aggregation and summarization of variant pathogenicity in genomic features of interest, e.g. counting the number of pathogenic variants in exonic regions.

### 2.3 Integration with variants of clinical significance

AlphaMissenseR implements functionality to contrast AlphaMissense predictions with variants of established clinical significance from the ClinVar database (Fig. 1C). ClinVar provides pathogenicity classifications for human genetic variants based on different clinical evidence categories (Landrum *et al.* 2018). The package provides an integrated analysis-ready dataset, derived from the supplemental data of the AlphaMissense publication (Cheng *et al.* 2023), that lists AlphaMissense pathogenicity scores alongside binary ClinVar classifications (benign/pathogenic) for 82k human variants across 7.8k proteins. Stored in a DuckDB database backend, this table can be used to efficiently query, visualize, and explore individual variants and genomic features of interest, and can be leveraged to evaluate and calibrate the predictions generated by AlphaMissense and other variant effect prediction models.

### 2.4 Benchmarking across proteins and models

For systematic and reproducible benchmarking of existing and new variant effect prediction methods, AlphaMissenseR integrates with ProteinGym (Fig. 1D), a comprehensive set of benchmarks specifically designed for protein fitness prediction (Notin *et al.* 2023). ProteinGym encompasses (i) a curated collection of over 250 standardized deep mutational scanning assays (DMS) profiling fitness effects of >2.5M mutations in 186 human proteins, and (ii) performance metrics for >60 leading variant effect prediction models. DMS experiments systematically measure the effects of all possible amino acid substitutions on the fitness of a protein (Fowler and Fields 2014), therefore providing suitable ground truth for evaluating variant effect predictions. For DMS assays of individual proteins, AlphaMissenseR computes Spearman correlation to contrast predicted pathogenicity with experimentally derived fitness scores, where a stronger negative correlation corresponds to a tighter relationship between the two measures. For comparative benchmarking of multiple variant effect prediction tools across the compendium of DMS assays, the package also provides pre-computed performance metrics derived from the DMS data for over 60 leading models.

### 2.5 Multi-level visualization and interactive exploration

AlphaMissenseR provides interactive exploration of variant pathogenicity at different levels and scales, allowing the user to interrogate the genomic and structural context of individual missense substitutions in a genome browser view or a 3D protein structure view (Fig. 1E). On the protein level, AlphaMissense pathogenicity scores can be projected onto interactive 3D protein structures using functionality from the r3dmol package (Su and Johnston 2022). On the gene level, variant effect prediction data stored in GenomicRanges objects can be explored by genomic location and pathogenicity classification in genome-browser widgets with Gosling (L’Yi *et al.* 2022). This facilitates the visual identification of areas of concentration of potentially pathogenic mutations, which could be regions of interest for further investigation, especially when overlaid with functional annotation such as protein domains or evolutionary conserved regions.

## 3 Conclusion

The AlphaMissenseR package provides a robust and reproducible framework for accessing, analyzing, and benchmarking missense variant pathogenicity predictions from AlphaMissense within the R/Bioconductor ecosystem. Leveraging a DuckDB backend, it enables high-performance querying of data from Zenodo with minimal memory use and integrates seamlessly with Bioconductor tools for genomic analysis and annotation. AlphaMissenseR supports comparisons with ClinVar variants of clinical significance and systematic benchmarking of prediction methods using data from 200+ deep mutational scanning assays and scores from 60+ tools. Taken together, AlphaMissenseR enables targeted investigations of missense mutations in genomic features of interest, and implements an extensible framework for the systematic and reproducible benchmarking of new and existing variant effect prediction tools.

## Data Availability

AlphaMissense data resources are distributed under the CC-BY 4.0 license and the AlphaMissenseR package is available from Bioconductor (https://bioconductor.org/packages/AlphaMissenseR) under the Artistic 2.0 license.
